# Factors Influencing Surgical Site Infections in a Tertiary Care Hospital: A Prospective Analysis

**DOI:** 10.7759/cureus.72767

**Published:** 2024-10-31

**Authors:** Shanmukha Koppolu, Gaurav Mittal, Shoraf Pascal, Yash Variya Takodara, Amit Ajay Singh

**Affiliations:** 1 Trauma and Orthopaedics, Barts Health NHS Trust, London, GBR; 2 Internal Medicine, Mahatma Gandhi Institute of Medical Sciences, Wardha, IND; 3 Research and Development, Student Network Organization, Mumbai, IND; 4 Community Medicine, Madha Medical College and Research Institute, Chennai, IND; 5 Surgery, Government Medical College, Alibag, Alibag, IND; 6 Surgery, Government Medical College, Latur, Latur, IND

**Keywords:** exploratory laparotomy, patient outcomes, prophylactic antibiotics, risk factors, surgical site infections

## Abstract

Background

Surgical site infections (SSIs) are a significant concern in surgical practice, contributing to increased morbidity, prolonged hospital stays, and healthcare costs. Understanding SSIs' incidence and risk factors is crucial for improving patient outcomes. This study aimed to identify the various risk factors contributing to SSIs in a tertiary hospital, with the goal of informing better clinical practices and preventive measures.

Methodology

This hospital-based, prospective observational study was conducted in the Department of General Surgery at Madha Medical College and Research Institute, Kanchipuram District, Chennai, India. Ethical approval was obtained, and patients aged 18 to 70 years who underwent non-traumatic exploratory laparotomy and developed SSIs were included. Data on socio-demographic characteristics, co-morbidities, surgical details, and postoperative outcomes were collected. Wound infections were diagnosed based on established clinical criteria, and microbiological analyses were performed on wound swabs.

Results

Out of 100 patients, 60 (60%) developed superficial SSIs, 30 (30%) had deep infections, and 10 (10%) experienced organ/space infections. Significant risk factors identified included prolonged surgical duration (55 patients, 55% affected), pre-existing diabetes (40 patients, 40%), and inadequate prophylactic antibiotic use (20 patients, 20%). The study reported an overall recovery rate of 95% among those treated for SSIs.

Conclusion

The study emphasizes the multifactorial etiology of SSIs, which leads to improved outcomes linked to SSIs, expanding preoperative assessment and suitable preventative measures before surgery, such as antibiotic prophylaxis.

## Introduction

Surgical site infections (SSIs) are an important healthcare issue concerning patients undergoing surgical intervention. These are the most common nosocomial infections among surgical patients and one of the major causes of nosocomial-associated morbidity and mortality. SSIs have also been regarded as one of the chief causes of adverse events due to surgical interventions, hence influencing the outcome of the patient [1,2]. The burden of SSIs tends to be higher in developing countries compared to developed regions, largely due to differences in healthcare infrastructure, infection control practices, and resource availability. A follow-up examination of scheduled surgical procedures classified as sterile and slightly contaminated surgeries conducted in 2019 reported an alarming 15% incidence of SSIs in low- and middle-income countries (LMICs) [3]. In the case of patients with SSIs, numerous complications are manifested, including long-term hospitalization, longer periods of recovery, and slow healing of wounds. Patients also endure more pain and anguish and, in extreme cases, permanent disability and death [4].

These infections also place a substantial financial burden on healthcare systems, as they extend patient care durations and necessitate more cost-intensive treatments. Several risk factors contribute to the development of SSIs, affecting them in complex patterns. Such factors include surgery-based patient factors, health condition-based patient factors, microbial features, and environmental factors [5]. This risk is greatly affected by various factors, which can encompass underlying diseases, the longer duration and type of surgery, the degree of wound contamination, and some patient-related factors like age, nutritional status, or immune function. Other contributory factors include malignancy, metabolic diseases, smoking, infections at distant sites, and even long preoperative periods in the hospital [6]. The challenge with SSIs is their persistent nature, highlighting the need for a thorough understanding of the risk factors associated with infection.

The most important ways to reduce the incidence of SSIs could be by identifying risk factors for individual patients and bridging gaps in present strategies for infection prevention. In all, this would result in a reduction in morbidity and mortality rates as well as a decrease in the financial burden on healthcare organizations. The present study focused on identifying the various risk factors contributing to SSIs in a tertiary hospital and will be useful in informing better clinical practice and preventive measures.

## Materials and methods

Study design and setting

The study was a hospital-based, prospective observational study carried out for a period of three months at the Department of General Surgery in Madha Medical College and Research Institute, Kanchipuram District, Chennai, India. Ethical approval for the study was granted by the institutional ethics committee. The study enrolled the patients who developed SSIs following non-traumatic exploratory laparotomies performed during the study period. The study was undertaken for three months, from April 2023 to June 2023. Consent was either obtained from or waived for all participants in this study. Madha Medical College and Research Institute issued approval (Approval No.: MMCRI/IEC/2023/087; dated: 27/02/2023).

Selection criteria

Patients were selected for this study based on specific inclusion and exclusion criteria. The inclusion criteria encompassed individuals aged 18 to 70 years, of either gender, who underwent non-traumatic exploratory laparotomy and developed SSIs afterward. Additionally, only patients willing to participate in the study were considered. The exclusion criteria ruled out patients undergoing laparotomy due to trauma, those receiving steroids, chemotherapy, radiotherapy, or immunosuppressive drugs, as well as individuals with prior skin infections or individuals who underwent surgery at external facilities.

Data sources and variables

The research was communicated to the participants in their native language, and written consent was secured for their involvement in the study. Information was collected on socio-demographic details, co-existing conditions such as diabetes mellitus, high blood pressure, asthma, thyroid conditions, kidney diseases, or any disorders related to immune suppression. Medical information was also recorded, including the use of prophylactic antibiotics, blood transfusions, length of preoperative hospital stay, American Society of Anesthesiologists (ASA) classification [6], characteristics of the surgical procedure, anesthesia administered, length of the operation, intraoperative observations, recovery process, present examination outcomes, and standard tests, including complete blood count (CBC), glucose levels, wound culture and sensitivity, liver function tests (LFT), and renal function tests (RFT).

A diagnosis of wound infection was made if any of the following criteria were met: serous or non-purulent discharge from the wound, pus discharge, serous or non-purulent discharge accompanied by signs of inflammation, or cases where the surgeon intentionally incised the wound to address localized fluid accumulation. Samples from the wound were forwarded to the microbiology lab for culture and sensitivity analysis. Following the findings of the culture and sensitivity results, the antibiotic regimen was adjusted if necessary. Patients were followed up until the SSI was fully resolved.

Statistical analysis

Data collection and compilation were done using Microsoft Excel (Microsoft Corporation, Redmond, WA), and statistical analysis was conducted using SPSS version 23.0 (IBM Corp., Armonk, NY), employing descriptive statistics. The descriptive analysis, like frequencies in terms of percentages, was calculated. Mean and standard deviations were also calculated for parameters like age.

## Results

Table 1 shows the demographic and clinical characteristics of the study participants. Of the 100 patients, 62 were male (62%) and 38 were female (38%), with a mean age of 45.6 years. Thirty patients (30%) had diabetes, while 24 (24%) had hypertension, and eight (8%) had bronchial asthma. Other comorbidities included thyroid disorders in five patients (5%) and renal disease in three patients (3%). Additionally, 80 patients (80%) received prophylactic antibiotics, and 42 patients (42%) had a preoperative hospital stay of more than five days, which could have contributed to an increased risk of SSIs.

**Table 1:** Demographic and clinical characteristics of study participants. The age has been depicted in the form of mean and standard deviation whereas other demographics have been depicted in the form of n (%), where "n" is the absolute number of patients.

Characteristic	Number of patients (%)
Total patients	100
Age (mean ± standard deviation)	45.6 ± 12.3
Gender	
Male	62 (62%)
Female	38 (38%)
Comorbidities	
Diabetes mellitus	30 (30%)
Hypertension	24 (24%)
Bronchial asthma	8 (8%)
Thyroid disorders	5 (5%)
Renal disease	3 (3%)
Prophylactic antibiotic use	80 (80%)
Preoperative hospital stay (>5 days)	42 (42%)

Table 2 highlights the surgical and intraoperative details of the participants, including the American Society of Anesthesiologists (ASA) classifications and anesthesia types. Forty patients (40%) were classified as ASA I, 35 patients (35%) as ASA II, and 25 patients (25%) as ASA III, indicating varying levels of preoperative health status. Seventy-five patients (75%) underwent surgery under general anesthesia, while 25 (25%) had spinal anesthesia. Fifty-five surgeries (55%) lasted for more than two hours, which is an important risk factor for SSIs. In terms of intraoperative findings, 78 patients (78%) had no complications, while 12 patients (12%) had adhesions, and 10 patients (10%) experienced bowel perforations, both of which could complicate the postoperative course and increase the risk of infection.

**Table 2:** Surgical and intraoperative details of study participants. ASA: American Society of Anesthesiologists. The parameters have been depicted as n (%), where "n" is the absolute number of patients.

Characteristic	Number of patients (%)
ASA score	
ASA I	40 (40%)
ASA II	35 (35%)
ASA III	25 (25%)
Type of anesthesia	
General anesthesia	75 (75%)
Spinal anesthesia	25 (25%)
Duration of surgery (>2 hours)	55 (55%)
Intraoperative findings	
No complications	78 (78%)
Adhesions	12 (12%)
Bowel perforation	10 (10%)

Table 3 focuses on the postoperative course and characteristics of SSIs among the study participants. Superficial SSIs were the most common, affecting 60 patients (60%), while 30 patients (30%) had deep SSIs, and 10 patients (10%) had organ/space infections. Regarding microbiological findings, 45 infections (45%) were caused by gram-positive bacteria, 40 (40%) by gram-negative bacteria, and 15 (15%) involved mixed infections. The initial antibiotic regimen was changed for 40 patients (40%) based on culture and sensitivity results. Fifty patients (50%) had a postoperative hospital stay exceeding seven days, and 95 (95%) fully recovered from their SSIs after follow-up.

**Table 3:** Postoperative course and SSI characteristics. SSI: surgical site infection. The parameters have been depicted as n (%), where "n" is the absolute number of patients.

Characteristic	Number of patients (%)
Type of SSI	
Superficial SSI	60 (60%)
Deep SSI	30 (30%)
Organ/space SSI	10 (10%)
Wound swab culture results	
Gram-positive bacteria	45 (45%)
Gram-negative bacteria	40 (40%)
Mixed infections	15 (15%)
Antibiotic change after culture	40 (40%)
Length of postoperative stay (>7 days)	50 (50%)
Complete recovery from SSI	95 (95%)

## Discussion

The present study's findings give an overall view of the occurrence of SSIs and risk factors, in agreement with global trends in infections among surgical patients. SSIs have been identified as one of the most common nosocomial infections in surgical wards; they greatly contribute to morbidity, prolong hospital stays, and increase healthcare costs. It has been estimated that SSIs occur in about 2-5% of patients undergoing surgery, thus posing a great danger to the safety of patients [7]. The World Health Organization also recognizes SSIs as a leading cause of postoperative complications by placing greater and more awareness on measures of implementing prevention strategies that include either surgical safety checklists or protocols concerning their occurrence [8]. In this study, most of the patients were infected with superficial SSI, which accounted for 60% of the total, whereas deep SSI and organ/space infections occurred in 30% and 10% of patients, respectively. Most of these infections are superficial and align with previous studies showing that gram-positive organisms, particularly *Staphylococcus aureus*, are commonly responsible [9]. The decolonization strategies combined with prophylactic antibiotic use are well-received in reducing gram-positive infections in surgical patients, especially in high-risk surgeries like orthopedics or cardiac surgeries [10].

The study also identifies various important risk factors for developing SSIs. One of the main risk factors is an average surgical time of over 100 minutes, documented in 55 patients (55%). Among them, 64% or 35 had this type of infection. The current study aligns with previous research on the view that longer operative times imply increased risks for infections, which occur when operations are extended, thereby increasing exposure to potential contaminants and reducing oxygenation of tissues [11]. It is important to note that while this association is evident, the observational nature of the study limits the ability to draw definitive causal conclusions. Patients with existing comorbid conditions, primarily diabetes mellitus, had a significantly increased rate of developing SSIs, whereby 40 out of every 100 patients, or 40%, suffered this complication, consistent with other reports that have indicated how diabetes severely hampers healing and predisposes patients to infection [12,13]. Another factor of great importance in terms of infection rate was the use of prophylactic antibiotics. In this study, 80 patients (80%) received prophylactic antibiotics; however, 90% of the patients (18 in number) who did not receive them developed SSIs. Therefore, strict adherence to evidence-based guidelines for antibiotic prophylaxis by different national and international bodies is called for [14,15]. The efficiency of timely and appropriate prophylaxis in decreasing the burden of SSIs is effectively shown in many studies [16,17]. This study's findings suggest that adherence to such guidelines may be beneficial, although further research is needed to establish a more causal relationship.

A prolonged preoperative stay was also found to be associated with SSIs, with 67% of cases who spent more than five days in the hospital before surgery also developing infections. This is comparable with other studies that show that the longer a patient spends time in the hospital, the higher their chances of contracting most of the infection-causing pathogens, increasing the risk of SSIs [18]. More so, the study affirms the evidence of systematic reviews showing that some of the most important risk factors are related to prolonged hospital stay and surgical duration [19]. This study showed that 45 patients were infected with gram-positive organisms, 40 with gram-negative organisms, and 15 had mixed infections. These are within the epidemiology of SSIs since gram-positive organisms, especially *Staphylococcus aureus* and *Staphylococcus epidermidis*, are the most common pathogens [20]. Nonetheless, it would not be rare to find gram-negative organisms like *Escherichia coli* and *Pseudomonas aeruginosa*, especially in abdominal surgeries [21].

Although the overall recovery rate in this study was promising at 95 out of 95 patients (95%), it necessitates the ongoing follow-up of the patients with SSIs. Proper postoperative surveillance, coupled with timely interventions such as adjustment of antibiotics based on culture and sensitivity reports, contributes to better outcomes for the patient. The study also reiterates the necessity of risk factors, which include pre-existing comorbid conditions, prolonged hospital stays, and the lack of usage of prophylactic antibiotics in low SSI rates amongst surgical patients [22]. In essence, this research shows the multifactorial nature of SSIs, meaning that specific targeted preventive interventions like antibiotic prophylaxis and timely surgical procedures, among other infection control approaches, play a key role. These risk factors may mitigate the incidence of SSIs substantially, thus improving patient outcomes and lowering healthcare costs associated with such infections [23].

Strengths and limitations of the study

This study is noteworthy for several reasons. Firstly, it provides a comprehensive analysis of the incidence and risk factors for SSIs in a tertiary care setting, contributing valuable data to the existing literature. The identification of significant associations between prolonged surgical duration and pre-existing comorbidities with SSI incidence highlights the need for targeted interventions. Additionally, the study's robust sample size and the focus on a range of surgical procedures enhance its applicability to clinical practice. Lastly, the findings underscore the importance of adherence to prophylactic antibiotic guidelines, which can play a crucial role in reducing SSI rates and improving patient outcomes.

The study has various limitations that cannot be ignored. It was conducted in only one facility, which may limit the generalizability of the findings to other contexts or populations. Additionally, the sample size, while adequate for pilot research, may not comprehensively represent all factors contributing to SSIs. The use of clinical diagnoses for SSIs, rather than more rigorous microbiological-based definitions, may impact the classification of infections. Moreover, the absence of microbiological analysis in determining culture and sensitivity patterns restricts the ability to correlate specific pathogens with SSIs and their management. Thirdly, the observational research design may have introduced selection bias and data collection issues that could influence the study's outcomes. Furthermore, unmeasured confounding variables, such as socioeconomic factors and hospital environmental conditions, were not investigated, which could provide further insights into the epidemiology of SSIs. Future studies with larger sample sizes and a more comprehensive approach to data collection, including microbiological analysis and statistical testing for associations, are needed to address these limitations effectively.

## Conclusions

In conclusion, the study is valuable in giving insights into the incidence and risk factors associated with SSIs after non-traumatic exploratory laparotomy. Results have thus emphasized superficial infection incidence, the influence that the prolonged duration of the surgical procedure exerts upon the result, and the presence of pre-existing comorbidities as predisposing factors to infection. The outcomes clearly emphasize adherence to established guidelines for prophylactic antibiotic use and the rigor of preoperative assessment to identify high-risk patients. Further, strict postoperative follow-up and timely intervention based on microbiological findings should also be directed to improve the outcome of patients. Since SSIs are multifactorial, an integrated approach would be required for their prevention as a multidisciplinary team effort from surgical teams to infection control practitioners and microbiologists.
